# Spider Venom Peptide Pn3a Inhibition of Primary Afferent High Voltage-Activated Calcium Channels

**DOI:** 10.3389/fphar.2020.633679

**Published:** 2021-01-28

**Authors:** Jeffrey R. McArthur, Nehan R. Munasinghe, Rocio K. Finol-Urdaneta, David J. Adams, Macdonald J. Christie

**Affiliations:** ^1^ Illawarra Health and Medical Research Institute (IHMRI), University of Wollongong, Wollongong, NSW, Australia; ^2^ Discipline of Pharmacology, University of Sydney, Sydney, NSW, Australia; ^3^ Electrophysiology Facility for Cell Phenotyping and Drug Discovery, IHMRI, Wollongong, NSW, Australia

**Keywords:** antinociceptive, calcium channel, dorsal root ganglion, high-voltage activated, opioids, pain, spider venom-derived peptide

## Abstract

Despite potently inhibiting the nociceptive voltage-gated sodium (Na_v_) channel, Na_v_1.7, *µ*-theraphotoxin Pn3a is antinociceptive only upon co-administration with sub-therapeutic opioid agonists, or by itself at doses >3,000-fold greater than its Na_v_1.7 *IC*_*50*_ by a yet undefined mechanism. Na_v_ channels are structurally related to voltage-gated calcium (Ca_v_) channels, Ca_v_1 and Ca_v_2. These channels mediate the high voltage-activated (HVA) calcium currents (*I*_*Ca*_) that orchestrate synaptic transmission in nociceptive dorsal root ganglion (DRG) neurons and are fine-tuned by opioid receptor (OR) activity. Using whole-cell patch clamp recording, we found that Pn3a (10 µM) inhibits ∼55% of rat DRG neuron HVA-*I*_*Ca*_ and 60–80% of Ca_v_1.2, Ca_v_1.3, Ca_v_2.1, and Ca_v_2.2 mediated currents in HEK293 cells, with no inhibition of Ca_v_2.3. As a major DRG *I*_*Ca*_ component, Ca_v_2.2 inhibition by Pn3a (*IC*_*50*_ = 3.71 ± 0.21 µM) arises from an 18 mV hyperpolarizing shift in the voltage dependence of inactivation. We observed that co-application of Pn3a and µ-OR agonist DAMGO results in enhanced HVA-*I*_*Ca*_ inhibition in DRG neurons whereas co-application of Pn3a with the OR antagonist naloxone does not, underscoring HVA channels as shared targets of Pn3a and opioids. We provide evidence that Pn3a inhibits native and recombinant HVA Ca_v_s at previously reportedly antinociceptive concentrations in animal pain models. We show additive modulation of DRG HVA-*I*_*Ca*_ by sequential application of low Pn3a doses and sub-therapeutic opioids ligands. We propose Pn3a's antinociceptive effects result, at least in part, from direct inhibition of HVA-*I*_*Ca*_ at high Pn3a doses, or through additive inhibition by low Pn3a and mild OR activation.

## Introduction

Physiological pain (acute, nociceptive) functions as early warning to protect the organism from injury. In contrast, pathological pain (chronic, neuropathic) originates from maladaptive operation of the nervous system. The sensory nervous system (primary afferent neurons, spinal interneurons, ascending tracts, and supraspinal areas) mediates pain signaling to the brain. Upon damage, nociceptors become sensitized/activated leading to opening of excitatory voltage-gated sodium (Na_v_) and calcium (Ca_v_) channels causing the subsequent firing of action potentials along sensory axons to the spinal cord. Induction and maintenance of central sensitization depend on peripheral nociceptors regarded as important targets for analgesics, with most efforts dedicated to the development of peripheral Na_v_1.7, Na_v_1.8, and Na_v_1.9 modulators as potential therapeutics.

Loss-of-function mutation of the *SCN9A* gene that codes for Na_v_1.7 leads to congenital insensitivity to pain (Cox et al., 2006), whereas *SCN9A* gain-of-function mutations causes paroxysmal extreme pain disorder and primary erythromelalgia (Dib-Hajj et al., 2008). These observations lead to increased interest in discovery of a highly selective and potent inhibitors of Na_v_1.7 to reduce the side effects seen in pan Na_v_ channel inhibitors. However, Na_v_1.7 inhibitors have for the most part failed to reproduce the pain-free state observed in chronic insensitivity to pain (CIP) (Eagles et al., 2020). Subsequently it was shown that genetic deletion of *SCN9A* in both mice and humans, that the absence of functional Na_v_1.7 but not Na_v_1.8, increases endogenous opioid receptor (OR) analgesia via upregulation of the enkephalin precursor *Penk* mRNA, which could be inhibited by the OR antagonist, naloxone (Minett et al., 2015). *µ*-Theraphotoxin Pn3a (μ-TRTX-Pn3a), a three disulphide bridged, 35 amino acid peptide isolated from the venom of the South American tarantula *Pamphobeteus nigricolour*, is a selective and potent Na_v_1.7 channel inhibitor (Deuis et al., 2017). Yet, despite Pn3a's high potency against Na_v_1.7-mediated currents, administration of 3 mg/kg of the peptide fails to produce analgesia in various animal pain models (Deuis et al., 2017). Nevertheless, local (3 µM) or systemic treatment (3 mg/kg) Pn3a administration causes antinociceptive behavior in mouse models of acute postsurgical pain. Interestingly, co-administration of Pn3a (1 mg/kg) with sub-therapeutic doses of opioids results in significant analgesia/anti-allodynia advocating for synergistic analgesic effects in rodent models of pain (Deuis et al., 2017; Mueller et al., 2019). In these reports, opioid receptor (OR) modulators naloxone and oxycodone did not alter Na_v_1.7 currents, nor Pn3a affected µ-/δ-/κ- OR mediated signaling (Mueller et al., 2019). Thus, the mechanisms behind the observed antinociceptive effects seemed unclear.

Animal toxins interacting with voltage-gated ion channels (VGICs) typically fall into two groups: pore blocking toxins, which sterically and electrostatically inhibit ion permeation (Hui et al., 2002; Finol-Urdaneta et al., 2020), and gating modifiers, that interact with the voltage sensor domain (Swartz and MacKinnon, 1995), such as Pn3a (Mueller et al., 2020). Recently, more detailed characterization of toxins and drugs once thought “selective” for a particular ion channel have proven active against others. Pore blocking Na_v_ channel toxins cross-react with other targets, include tetrodotoxin (Na_v_/Ca_v_), saxitoxin (Na_v_/K_v_/Ca_v_) and *µ*-conotoxin PIIIA (Na_v_/K_v_/Na_v_Bac) (McArthur et al., 2011; Leipold et al., 2017; Finol-Urdaneta et al., 2019); whereas voltage-sensing domain (VSD) gating modifier toxins like ProTxI (Na_v_/K_v_/Ca_v_/TRPA1) (Middleton et al., 2002; Bosmans et al., 2008; Bladen et al., 2014; Gui et al., 2014), ProTxII (Na_v_/Ca_v_) (Middleton et al., 2002; Bladen et al., 2014), kurtoxin (Na_v_/Ca_v_) (Chuang et al., 1998), Hanatoxin (K_v_/Na_v_/Ca_v_) (Swartz and MacKinnon, 1995; Li-Smerin and Swartz, 1998; Bosmans et al., 2008), and JZTX-I (Na_v_/K_v_) (Xiao et al., 2005; Yuan et al., 2007). Furthermore, small molecule compounds modulating VSDs include capsaicin, and capsazepine (TRPV1/K_v_/Ca_v_) (Kuenzi and Dale, 1996; Caterina et al., 1997; McArthur et al., 2019) to name a few.

Given the structural conservation between Na_v_s and Ca_v_s, the actions of Pn3a over native and recombinant Ca_v_ channels were investigated. Whole-cell patch clamp recording was used to assess Pn3a's effects on high voltage-activated (HVA) calcium currents (*I*_*Ca*_) in rat DRG neurons and heterologously expressed Ca_v_ channels in HEK293 cells. Our results show that Pn3a inhibits HVA *I*_*Ca*_ in isolated rat DRG neurons at concentrations reported to produce analgesia. Importantly, Pn3a's inhibitory effect over *I*_*Ca*_ was additive to that of sub-therapeutic opioid receptor agonists highlighting concerted inhibition of HVA by Pn3a/opioids as a potential component of the antinociceptive effects observed in animal pain models.

## Methods

### Dorsal Root Ganglion Isolation and Culture

Rats were purchased from the Animal Resources Center (Perth, Australia; 3–5 weeks old male Sprague Dawley rats) kept in groups of four, on a 12 h–12 h light–dark cycle at 22 ± 2°C with environment enrichment inside individually ventilated cages. Food and water were provided *ad libitum*. In total, DRG neurons from 30 rats were used in this study. To extract neurons, rats were decapitated under anesthesia with 4% isoflurane in the air. Experiments were conducted under the project number 2015/830 approved by the Animals Ethics Committee (AEC) at the University of Sydney, NSW, Australia. AEC guidelines comply with the ‘Australian code of practice for the care and use of animals for scientific purposes’, the ARRIVE guidelines on reporting experiments involving animals.

Spinal level L3–L5 DRG were removed and placed in ice-cold HEPES-buffered saline (HBS) composed of (in mM): 154 NaCl, 2.5 KCl, 1.8 CaCl_2_, 1.5 MgCl_2_, 10 HEPES, and 10 glucose (pH 7.4, 330 ± 5 mOsm). Iridectomy scissors were utilized to cut up ganglia before incubation at 37°C for 15 min in oxygenated HBS containing 5 mg ml^−1^ collagenase type 2 (Worthington Biochemical Corp, Lakewood, NJ, United States) followed by 25 min in oxygenated HBS containing 1 mg ml^−1^ papain (Worthington Biochemical Corp). The enzyme activity was terminated with the addition of HBS containing a combination of 1 mg ml^−1^ bovine serum albumin (BSA) and 1 mg ml^−1^ trypsin inhibitor (Sigma). After enzyme treatment, ganglia were placed in 3 ml of HBS following two room temperature HBS washes. Ganglia were then triturated through fire-polished Pasteur pipettes with decreasing bores to extract cells. Finally, the cells mixed in HBS were plated onto surface modified culture dishes (Corning Primaria™ easy grip). Cells were viable at room temperature for approximately 8 h. DRG cells were pre-treated with 1 μg ml^−1^ Alexa Fluor 488-conjugated *Bandeiraea simplicifolia* IB_4_ (Invitrogen) for 5 min at room temperature and washed with HBS for 5 min before fluorescence was examined on the inverted microscope (Olympus, IX50) used for patch clamp recordings.

### Cell Culture and Transfection

Human embryonic kidney (HEK293T) cells containing the SV40 Large T-antigen were cultured at 37°C, 5% CO2 in Dulbecco’s Modified Eagle’s Medium (DMEM, Invitrogen Life Technologies, Australia), supplemented with 10% fetal bovine serum (FBS, Bovigen, Australia), 1% GlutaMAX and penicillin-streptomycin (Invitrogen, Australia). HEK293 cells were then transiently co-transfected with the different voltage-gated calcium channel isoforms and green fluorescent protein (GFP) for visualization, using the calcium phosphate method. cDNAs encoding mCa_v_1.2 (a gift from Dr. D. Lipscombe; Addgene plasmid #26572) (Helton et al., 2005), rCa_v_1.3 (a gift from Dr. D. Lipscombe; Addgene plasmid # 49,333) (Xu and Lipscombe, 2001), hCa_v_2.1 (a gift from Dr. J. Striessnig), rCa_v_2.2 (a gift from Dr. D. Lipscombe), hCa_v_2.2 (a gift from Dr. D. Yue), hCa_v_2.3 (purchased from GenScript United States Inc.) were co-transfected with β_3_, α_2_δ_1_ and GFP. After transfection, cells were plated on 12 mm cover glass and transferred to a 30°C incubator for 1–3 days.

### Electrophysiology of Native High Voltage-Activated (HVA) Calcium Currents

Cell size was determined from an eyepiece graticule. Whole-cell electrophysiology was conducted at room temperature (22–24°C) in culture dishes perfused with room temperature HBS. The intracellular pipette solution contained (mM): 120 CsCl, 10 HEPES, 10 EGTA, 2 CaCl_2_, 5 MgATP, 5 Na_2_GTP, 5 NaCl; pH 7.3 (CsOH); 285 ± 5 mos mol l^−1^. To isolate *I*_Ca_, the extracellular solution contained (mM): 140 tetraethylammonium chloride (TEA-Cl), 2.5 KCl, 1.8 CaCl_2_, 1.2 MgCl_2_, 10 HEPES, 10 glucose; pH 7.4 (CsOH); 330 ± 5 mosmol. l^−1^. The liquid junction potential was 9 mV. The extracellular solution to isolate *I*_Na_ contained (mM): 110 TEA-Cl, 30 NaCl, 2.5 KCl, 1.8 CaCl_2_, 1.2 MgCl_2_, 10 HEPES, 10 D-glucose, 0.1 CdCl_2_, 0.1 mg ml^−1^ BSA; pH 7.2 (CsOH); 330 ± 5 mosmol. l^−1^.

An EPC-9 patch-clamp amplifier and corresponding PULSE software from HEKA Electronik (Lambrecht/Pfalz, Germany) were used to make recordings. Currents were sampled at 50 kHz and recorded on a hard disk. Borosilicate glass from AM Systems, Everett, WA, United States were used for patch pipettes. The input resistance of the pipettes ranged from 2 to 4 MΩ. The cell capacitance was between 7 and 20 pF (15–25 µm), while series resistance under 9 MΩ was deemed acceptable. All experiments had a series resistance compensation of at least 80%. A built-in procedure of the HEKA amplifier compensated for the capacitive transient currents. A −**P**/6 protocol was utilized to subtract leak current online. *I*_Ca_ was generated with the recording protocol which included a depolarization from a holding potential of −80 mV to 0 mV for 10 ms every 30 s. *I*_Na_ was generated through a recording protocol which included a step depolarization from a holding potential of −80 mV to 0 mV for 10 ms at a frequency of 0.1 Hz. Cells were perfused with the test compound via a pressure driven perfusion system (AutoMate Scientific, United States). Pn3a mediated inhibition was determined by measuring the decrease in peak *I*_Na_ or **I**_Ca_ amplitude from baseline upon perfusion of the compound.

### Electrophysiology of Transiently Transfected Ca_v_s

Depolarization-activated Ca^2+^ currents (I_*Ca*_) in transfected HEK293 cells were recorded in the whole-cell patch clamp configuration. Data were recorded using a MultiClamp 700B amplifier, digitized with a DigiData1440 and controlled using Clampex10.7 software (Molecular Devices, United States). Currents were sampled at 100 kHz and filtered to 10 kHz, with leak and capacitive currents subtracted using a −P/4 protocol. Extracellular recording solution contained as follows (mM): 100 NaCl, 10 CaCl_2_, 1 MgCl_2_, 5 CsCl, 30 TEA-Cl, 10 D-glucose and 10 HEPES, pH 7.3 with TEA-OH. Fire-polished borosilicate (1B150F-4, World Precision Instruments, Sarasota, FL, United States) patch pipettes were used with resistance of 1-3 MΩ and compensated > 80%. Intracellular recording solution contained as follows (mM): 140 KGluconate, 5 NaCl, 2 MgCl_2_, 5 EGTA and 10 HEPES, pH 7.2 with KOH. Cells were continuously perfused with extracellular solution at a rate of ∼1.2 ml/min. Depolarization-activated currents were elicited from a holding potential of −90 mV to a test potential (20 ms) for different channel isoforms determined by the peak amplitude elicited during an I-V protocol at a rate of 0.2 Hz Pn3a was superfused over the cell using a syringe pump. Activation curves of hCa_v_2.2 were generated through a series of test pulses (50 ms) ranging from −30 to +50 mV (Δ5 mV) every 5 s with a holding potential of −90 mV. Steady-state inactivation (SSI) curves for hCa_v_2.2 were generated by a series of pre-pulse potentials ranging from −120 to +30 mV of 1 s prior to a test pulse (50 ms) of +20 mV.

### Data and Statistical Analysis

All data analysis and graphs were generated in OriginPro (Origin Lab Corporation, United States). Concentration-response relationships were built by plotting peak current amplitudes in the presence of Pn3a (I_Pn3a_), over the current prior to Pn3a application (I_Control_). The resulting curve was fit with a sigmoidal curve according to the following expression:

$$I_{Pn3a}/I_{Control}=1+{\left[Pn3a\right]}^{n}/\left(IC50^{n}+{\left[Pn3a\right]}^{n}\right)$$

Where IC_50_ is the half-maximal inhibitory concentration and *n* is the Hill coefficient. Activation and SSI curves were fit by the modified Boltzmann equation:

$$I\;or\;G=1/\left(1+\exp\left(\frac{Vm-V_{0.5}}{ka}\right)\right)$$

Where I is the current or G is the conductance, *Vm* is the pre-pulse potential, *V*_*0.5*_ is the half-maximal activation potential and *ka* is the slope factor.

Statistical significance (*p* < 0.05) was determined using unpaired Student’s t-test or 1-way ANOVA followed by a Tukey multiple comparison. All data is presented as mean ± SEM (n), where n is individual cells.

### Materials

Naloxone hydrochloride and tetrodotoxin (TTX) were from Tocris (Bristol, United Kingdom) and solubilized in H_2_O. [D-Ala^2^, N-MePhe^4^, Gly-ol]-Enkephalin (DAMGO) was from Sigma-Aldrich (St. Louis, MO, United States) and solubilized in H_2_O. Synthetic µ-Theraphotoxin-Pn3a (UniProtKB - P0DM12) was kindly provided by Dr. Irina Vetter’s laboratory, Institute for Molecular Bioscience, University of Queensland and solubilized in H_2_O.

## Results

### Pn3a Reduces Rodent High Voltage-Activated Ca^2+^ Currents in Small Dorsal Root Ganglion Neurons

**Figure 1:**
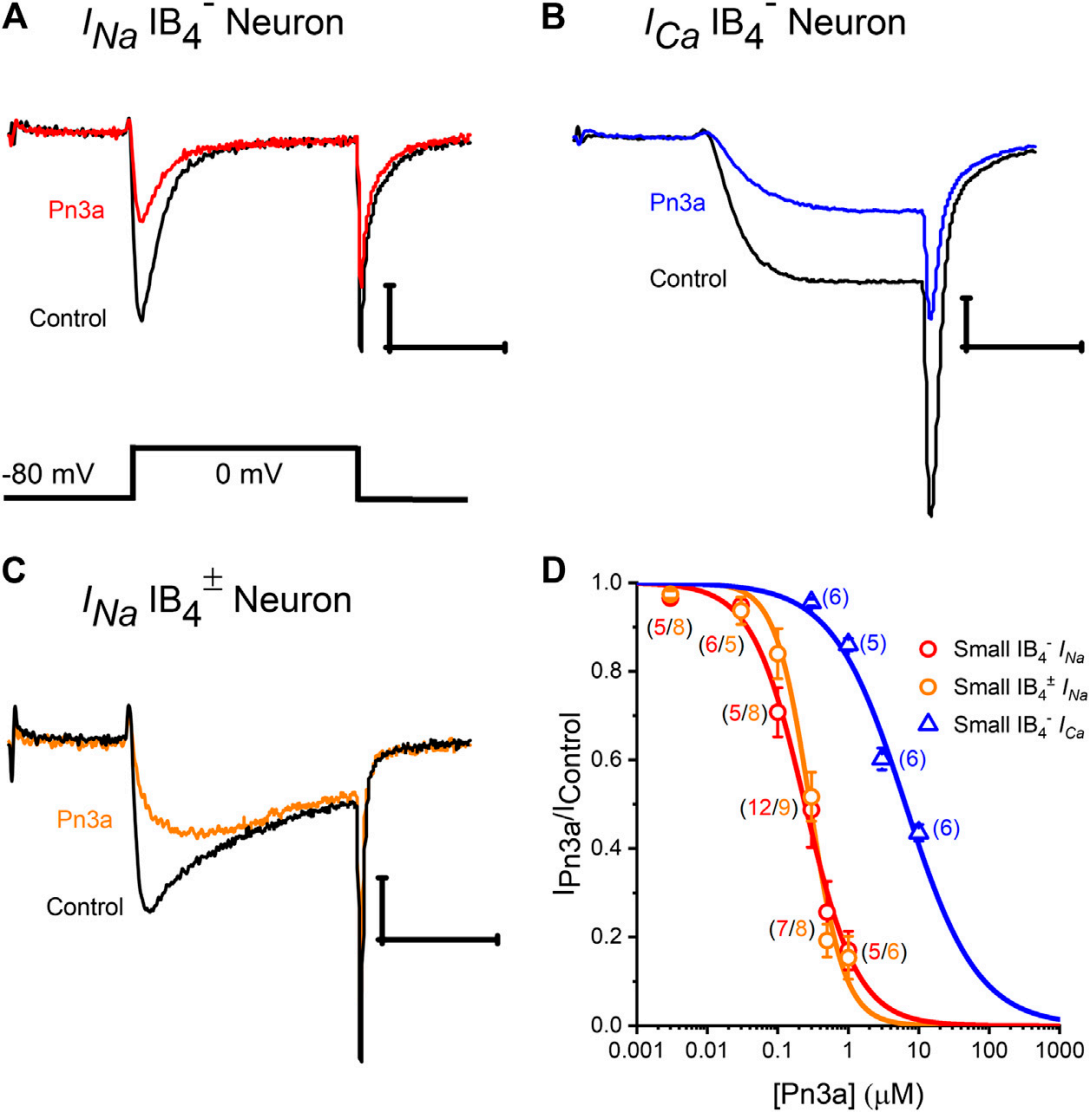
Pn3a inhibition of TTXs-*I*_*Na*_ and high voltage-activated *I*_*Ca*_ in rat DRG neurons. **(A–C)** Representative recording of control (black) I_*Na*_ in IB_4_^−^ and IB_4_^±^ DRG neurons (red and orange, respectively) in the presence of 0.3 µM Pn3a. **(B)** HVA *I*_*Ca*_ currents from DRG neurons in control (black) and the presence of 10 µM Pn3a (blue). **(D)** Concentration-response curves obtained for Pn3a inhibition of TTXs-*I*_*Na*_ in small-diameter IB_4_^−^ (red) and small-diameter (5–60 pF) lightly IB_4_^±^ (orange) neurons and *I*_*Ca*_ in small-diameter IB_4_^−^ (blue) neurons (n values shown for each concentration). X scale bar 5 msec; Y scale bar 0.5 nA.

Robust depolarization-activated sodium (*I*_*Na*_) and calcium (*I*_*Ca*_) currents can be elicited in rat small-diameter (7–20 pF) DRG neurons that were inhibited by application of Pn3a (Figure 1). Representative isolectin-B4 lightly positive (IB_4_^±^) and negative (IB_4_^−^) DRG neurons mediated *I*_*Na*_ and *I*_*Ca*_ whole-cell currents in control (black) and in the presence of Pn3a (colored) are shown in Figures 1A–C. To compare channel populations mediating TTXs-*I*_*Na*_ and HVA-*I*_*Ca*_ active during DRG neuron action potential firing, Pn3a exposure experiments were performed using identical pulse protocols (holding potential, −80 mV to a test potential of 0 mV, 10 ms). It can be appreciated that ∼50% of *I*_*Na*_ remained after application of 300 nM Pn3a in both small-diameter DRG neuron subtypes tested; whereas 10 μM Pn3a spared half of the HVA *I*_*Ca*_ observed in IB_4_^−^ DRG neurons. Concentration-response curves (CRC) were generated to compare the potency of Pn3a against *I*_*Na*_ and *I*_*Ca*_ in these neurons under our experimental conditions (Figure 1D).

Pn3a inhibition of (TTX-sensitive) TTXs-*I*_*Na*_ in IB_4_^−^ or IB_4_^±^ small-diameter DRG neurons had similar *IC*_*50*_s (0.24 ± 0.03 µM vs. 0.29 ± 0.02 µM, respectively), which were comparable to previously reported values (Deuis et al., 2017), and was fully reversible upon washout (Supplementary Figure S1). From these two neuronal subtypes, we concentrated on small-diameter IB_4_^−^ DRG neurons, as Pn3a displayed more potent block of *I*_*Ca*_ in this neuronal population with a calculated *IC*_*50*_ of 6.43 ± 0.53 µM. Thus, Pn3a inhibits HVA I_*Ca*_ known to be critical for neurotransmitter release and pain signal propagation (Bourinet et al., 2014).

### Pn3a Inhibits Most Ca_v_ Channel Isoforms That Mediate Afferent I_Ca_

**Figure 2:**
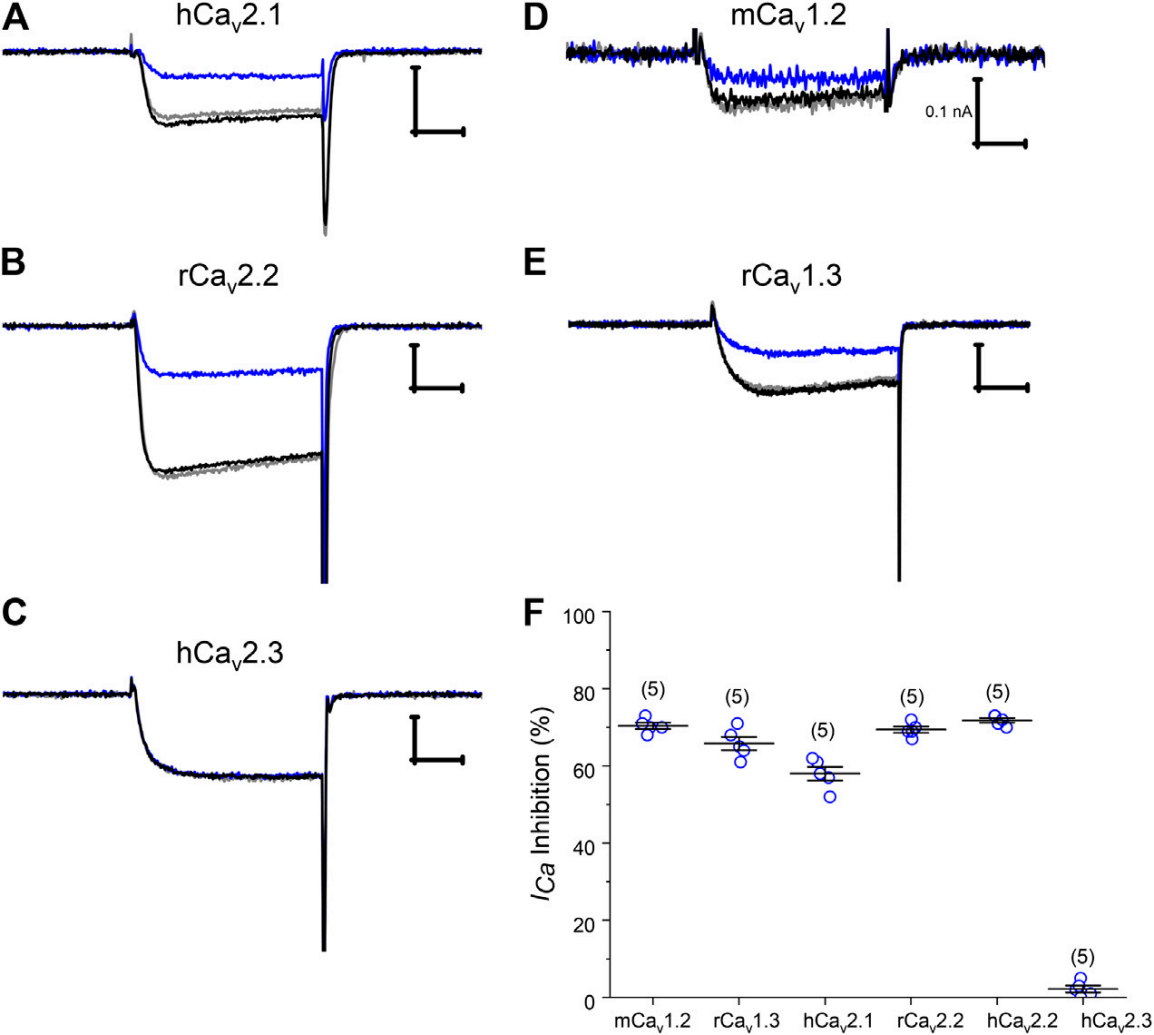
Pn3a modulation of recombinant HVA Ca_v_ channels. **(A–E)** Representative current traces of Ca_v_2.1, Ca_v_2.2, Ca_v_2.3, Ca_v_1.2, and Ca_v_1.3 mediated Ca^2+^ currents in control (black), in the presence of 10 µM Pn3a (blue), and after washout (gray). **(F)** Summary bar graph comparing 10 µM Pn3a inhibition (%) of heterologously expressed HVA calcium channels predominantly expressed in rodent DRG neurons. Mouse Ca_v_1.2, rat Ca_v_1.3, 2.2 and human Ca_v_2.1, 2.2, 2.3 channels were all co-expressed with human β_3_ and α_2_δ_1_ in HEK293 cells (n = 5 for each channel). X scale bar 5 msec; Y scale bar 0.5 nA, unless noted.

Numerous HVA Ca_v_ channel isoforms are present in rat DRG neurons. In order to ascertain the channel target underlying Pn3a's activity on neuronal HVA *I*_*Ca*_, Pn3a's activity was initially screened on a panel of representative HVA Ca_v_ channels typically expressed in DRG neurons including Ca_v_1.2, Ca_v_1.3, Ca_v_2.1, Ca_v_2.2, and Ca_v_2.3. We transiently co-transfected the individual Ca_v_ channel α-subunits of interest together with β_3_ and α_2_δ_1_ subunits in HEK293 cells and measured whole-cell *I*_*Ca*_ currents by patch clamp. *I*_*Ca*_ was elicited from a holding potential of −90 mV to a test potential of +20 mV for 20 ms, at a frequency of 0.2 Hz. A large component of *I*_*Ca*_ in rat DRG neurons is carried by Ca_v_2.2 channels therefore we began our screen testing the effects of 10 µM Pn3a on rat and human Ca_v_2.2 mediated currents (Figure 2). Both channel orthologues were similarly suppressed by 69.5 ± 1.0% and 71.8 ± 0.6% (*n* = 5, *per* orthologue), respectively. Other HVA calcium channels typically expressed in DRG neurons including mouse Ca_v_1.2, rat Ca_v_1.3, human Cav2.1, Ca_v_2.2, and Ca_v_2.3 were exposed to 10 µM Pn3a and further examined. All but one of the HVA channels studied were sensitive to Pn3a block (Figure 2) and fully reversible upon washout (Supplementary Figure S2). Specifically, inhibition of mCa_v_1.2-mediated currents reached 70.3 ± 1.5% (*n* = 5), rCa_v_1.3: 65.6 ± 1.8% (*n* = 5) and hCa_v_2.1: 58.0 ± 1.8% (*n* = 5). Interestingly, application of up to 10 µM Pn3a had negligible effects on hCa_v_2.3 mediated currents (2.2 ± 0.9%, *n* = 5) (Figures 2C,F). The level of Pn3a Ca^2+^ current suppression observed in the recombinant channels was consistent with our observations on HVA *I*_*Ca*_ in DRG neurons (56.5 ± 1.9%, *n* = 6) (Figure 1D), suggesting that compounded inhibition of HVA *I*_*Ca*_ by Pn3a may be due to its equipotent effects on multiple Ca_v_ channels rather than a particular isoform.

### Pn3a Produces a Hyperpolarizing Shift in Ca_v_2.2 Steady-State Inactivation

**Figure 3:**
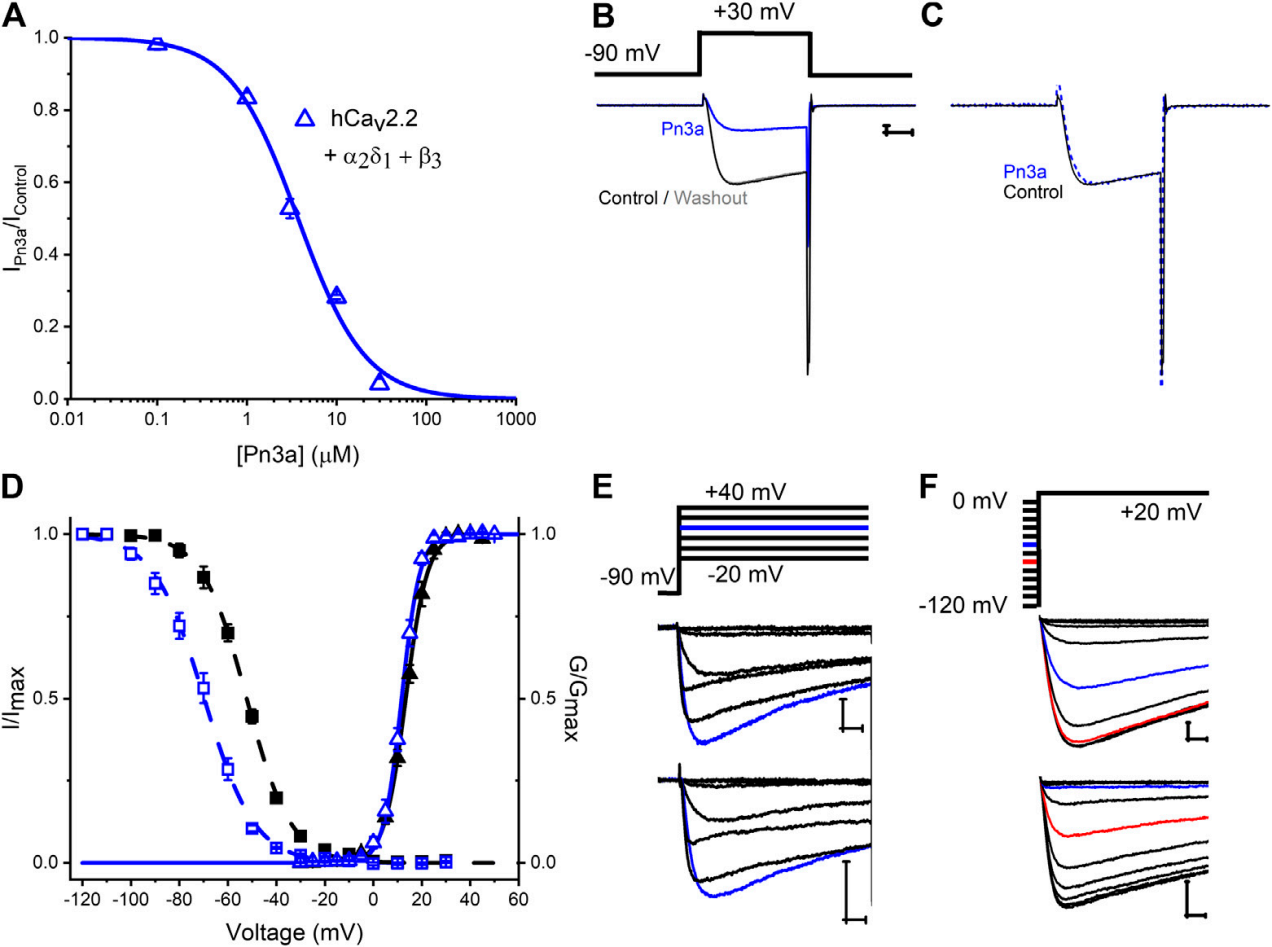
Characterization of Pn3a effects on Human Ca_v_2.2 channel-mediated currents. **(A)** Pn3a inhibition of hCa_v_2.2 currents is described by the concentration-response curve. Hill equation fit gives an *IC*_*50*_ = 3.70 ± 0.21 µM, and n_H_ = 1.16 ± 0.07 (*n* = 5 for each concentration). **(B)** Representative current traces of hCa_v_2.2 (β_3_/α_2_δ_1_) recorded in control (black), 10 µM Pn3a (blue), and after peptide washout (gray). **(C)** Normalized peak currents from **(B)** in control (black, solid) and Pn3a (blue, dotted). **(D)** Effect of Pn3a on the voltage dependent kinetics of Ca_v_2.2. Activation (triangles) and steady-state inactivation (squares) relationships for hCa_v_2.2 obtained in the absence (▲, ■) and presence of 3 µM Pn3a (**△**, ⊚). **(E)** Representative currents elicited by the voltage protocol **(top)** in the absence (control, middle) and presence of 3 µM Pn3A **(bottom)** normalized to peak I-V currents (blue trace, +20 mV) used to generate activation curves shown in **(D)**. **(F)** Representative currents from steady-state inactivation (SSI) protocol **(top)** obtained in the absence (control, middle) and presence of 3 µM Pn3a used to generate SSI curves shown in **(D)**. X scale bar 5 msec; Y scale bar 0.5 nA.

Ca_v_2.2 is regarded as a major contribution to HVA *I*_*Ca*_ in rat DRG neurons and is expressed at a similar level across different DRG neuron sizes/types (Scroggs and Fox, 1992) and, from our screening, a prominent Pn3a target. A concentration-response curve for inhibition of recombinant hCa_v_2.2 in the presence of Pn3a is shown in Figure 3A. The Pn3a CRC was accurately described by a Hill fit with an *IC*_*50*_ of 3.7 ± 0.2 μM, and **n**_H_ = 1.16 ± 0.07 (*n* = 5 *per* concentration). The mechanism of action of Na_v_-targeting therapotoxins often involves gating modification (Bosmans and Swartz, 2010), therefore, we investigated in detail the mechanism of inhibition of Pn3a over recombinant hCa_v_2.2 mediated Ca^2+^ currents. Depolarization-activated hCa_v_2.2 current kinetics were not altered in the presence of 10 µM Pn3a as observed in Figures 3B,C where control and toxin exposed currents have been scaled for ease of comparison. The activation voltage dependence of hCa_v_2.2 mediated *I*_*Ca*_ in the presence of 3 µM Pn3a was slightly shifted from control currents (Control V_0.5_ = 13.4 ± 0.1 mV, *ka =* 4.4 ± 0.1, and Pn3a V_0.5_ = 11.7 ± 0.1 mV, *ka* = 3.7 ± 0.1; *n* = 5; V_0.5_ *p* < 0.0001; Figures 3D,E). Ca_v_2.2 steady-state inactivation was evaluated with a standard protocol (Figure 3F) in control and during exposure to 3 μM Pn3a. In the presence of Pn3a, Ca_v_2.2 mediated currents displayed enhanced inactivation with a ∼18 mV hyperpolarizing shift in SSI (Control V_0.5_ = −52.3 ± 0.2 mV, *ka =* 9.2 ± 0.2, and Pn3a V_0.5_ = −69.8 ± 0.3 mV, *ka* = 10.5 ± 0.3; *n* = 5; V_0.5_ *p* < 0.0001). Hence, Pn3a appears to inhibit HVA Ca_v_2.2 by promoting entrance to the inactivated state.

### Rat Small IB_4_^−^ DRG Neurons HVA-I_Ca_ is Additively Inhibited by Pn3a and Low Doses of Opioids Agonists but Not Antagonists

**Figure 4:**
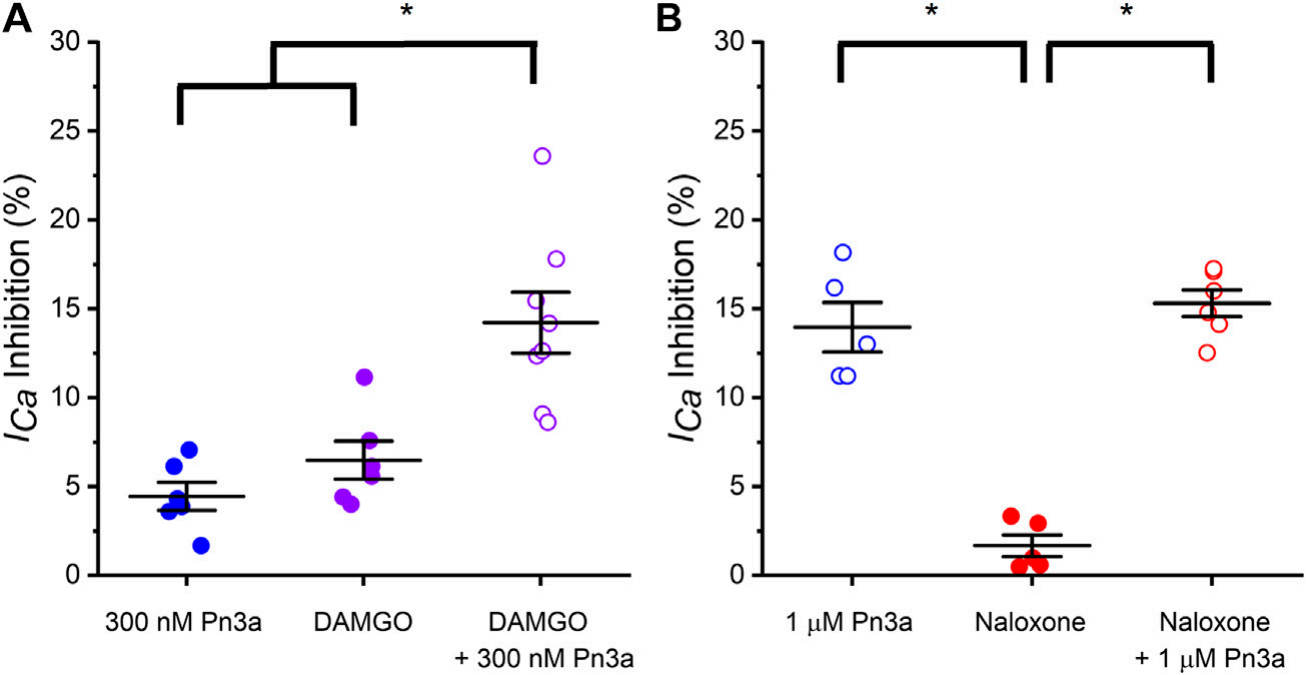
Pn3a and opioids inhibit rat DRG neurons high voltage-activated *I*_*Ca*_. **(A)** Bar graph summary of *I*_*Ca*_ modulation by OR agonist DAMGO and Pn3a in small IB_4_^−^ DRG neurons. 300 nM Pn3a (⊚; *n* = 6), 100 nM DAMGO (⊚; *n* = 6), 100 nM DAMGO +300 nM Pn3a (⊚; *n* = 8) **(B)** Bar graph summary of *I*_*Ca*_ modulation by OR antagonist DAMGO and Pn3a in small IB_4_^−^ DRG neurons. 1 µM Pn3a (⊚; *n* = 5), 1 µM naloxone (⊚; *n* = 5) and 1 µM Pn3a +1 µM naloxone (⊚; *n* = 6). One-way Anova results (* = *p* < 0.001).

To examine a potential contribution of HVA calcium channel inhibition by Pn3a to the reported synergy with opioids observed in animal pain models (Mueller et al., 2019), the effects of co-application of Pn3a and sub-therapeutic doses of opioid modulators DAMGO (OR agonist) and naloxone (OR antagonist) on HVA *I*_*Ca*_ from rat DRG neurons were assessed. Co-application of Pn3a (300 nM) and DAMGO (100 nM) resulted in a 14.22 ± 1.72% (*n* = 8; *p* < 0.001) (Figure 4A) reduction of the total HVA *I*_*Ca*_. This is significantly higher than the inhibition of HVA *I*_*Ca*_ achieved with the same concentrations of Pn3a (4.45 ± 0.78%, *n* = 6; *p* < 0.001) and DAMGO (6.49 ± 1.07%, *n* = 6; *p* > 0.001 applied independently (Figure 4A). These results are consistent with the additive inhibition of small-diameter DRG *I*_*Ca*_ by activation of ORs using DAMGO and Pn3a. In turn, the suppression of rat neuronal *I*_*Ca*_ by individual application of 1 µM Pn3a (13.97 ± 1.39%, *n* = 5) or co-application with 1 µM naloxone (15.31 ± 0.75%, *n* = 6) were undistinguishable, whilst on its own 1 µM naloxone minimally affected *I*_*Ca*_ (1.68 ± 0.61% inhibition, *n* = 5; *p* < 0.001) (Figure 4B).

## Discussion

This study explored the effects of the analgesic spider venom-derived peptide Pn3a on afferent HVA Ca^2+^ currents revealing that L-, P/Q, and N-type, but not R-type, Ca_v_ channels are all susceptible to inhibition by this VDP. Pn3a's mechanism of inhibition of Ca_v_2.2 channels was determined as the major isoform mediating HVA *I*_*Ca*_ in nociceptive DRG neurons. Finally, additive suppression of neuronal HVA *I*_*Ca*_ by Pn3a and OR agonist, DAMGO was verified.

### Pn3a Inhibits High Voltage-Activated Ca_v_ Channels

Pn3a is a potent (IC_50_ = 0.9 ± 0.8 nM) and selective Na_v_1.7 gating-modifier spider venom peptides targeting its DII VSD (Deuis et al., 2017). Homology of VGIC voltage sensing domains, and particularly those of Na_v_ channels, is at the crux of the development of genuinely selective Na_v_1.7 modulators (Kingwell, 2019). Given the VSD functional importance in neuronal VGICs and their structural homology, it is not surprising that VDPs target related channel families. This, together with Ca_v_ channels as downstream targets of opioid analgesic drugs (Hescheler et al., 1987), motivated our investigation of Pn3a's modulation of small-diameter DRG neuron HVA calcium channels.

Pn3a was shown to strongly suppress TTXs-*I*_*Na*_ in small diameter IB_4_^−^ DRG neurons and reduce c-fibre evoked excitatory post-synaptic currents. These neurons contain large HVA Ca^2+^ currents (Figure 1B), which could also contribute to the observed reduction in c-fibre excitatory post-synaptic currents (Deuis et al., 2017). We observed suppression of the HVA Ca^2+^ currents upon application of Pn3a with an IC_50_ value of 6.43 µM, a concentration ∼25-fold higher than that required to inhibit *I*_*Na*_ in small diameter IB_4_^−^ DRG neurons (Figure 1D). HVA *I*_*Ca*_ in these neurons is mediated by several Ca_v_ channel subfamilies, including L-(Ca_v_1.2 and Cav1.3 predominantly), P/Q- (Ca_v_2.1), N- (Ca_v_2.2), and R-type (Ca_v_2.3) (Fuchs et al., 2007). Our experiments with recombinant Ca_v_1 and Ca_v_2 channels clearly show their sensitivity to Pn3a modulation (Figure 2). Earlier work investigating Pn3a (10 µM) activity on endogenous calcium currents in SH-SY5Y cells relied on fluorescence-based (FLIPR) assays of KCl-induced changes in intracellular Ca^2+^ concentration in which the lack of voltage control precludes reliable Ca_v_ channel activation (Deuis et al., 2017) perhaps missing the Pn3a effects reported here with direct measurement of Ca_v_2.2-mediated currents.

Pn3a inhibits all the HVA Ca_v_ isoforms tested here with the notable exception of Ca_v_2.3, which was minimally affected by up to 10 µM Pn3a (Figure 2F). This observation highlights the presence of a conserved Pn3a-binding site in all HVA Ca_v_ channels but the R-type isoform. Future work is required to attempt to isolate Pn3a's binding site on HVA Ca_v_ channels, which could provide valuable information for future design of selective Na_v_1.7, or HVA calcium channel inhibitors, to attempt to dissect out VDP cross talk between these two channel families. A provocative possibility is that analgesic therapeutics may profit from Na_v_/Ca_v_ polypharmacology for which Pn3a may provide a valuable scaffold. This has been shown effective for other potential therapeutic compounds including CNCB-2, a dual Na_v_1.7/Ca_v_2.2 inhibitor that suppresses action potential firing in small diameter DRG neurons, providing long lasting analgesia in postoperative surgical pain and inflammatory pain models (Lee et al., 2019).

### Pn3a Inhibition of High Voltage-Activated Ca_v_s Differs From That of Na_v_s

Pn3a's inhibition of Na_v_1.7 current is concurrent with a depolarizing shift in the channel’s voltage dependence of activation without discernible effects on SSI. Pn3a gating modification of Na_v_1.7 currents is reported to arise from interactions with one or more Na_v_1.7 VSDs, as suggested by Pn3a inhibition of K_v_2.1-Na_v_1.7 (DII and DIV) VSD chimeras (Bosmans et al., 2008; Deuis et al., 2017). Mammalian Na_v_ and Ca_v_ α-subunits are structurally homologous but differ in their gating mechanisms (Kubota et al., 2017). Our analysis of Ca_v_2.2 inhibition by Pn3a revealed a hyperpolarizing shift in steady-state inactivation without apparent changes in channel activation (Figure 3D). Other known gating modifier toxins including Kurtoxin, Hanatoxin, Jingzhaotoxin-I, and Protoxin I/II inhibit both Na_v_ and Cav channels (Swartz and MacKinnon, 1995; Chuang et al., 1998; Li-Smerin and Swartz, 1998; Yamamoto and Sakashita, 1998; Middleton et al., 2002; Sidach and Mintz, 2002; Xiao et al., 2005; Bosmans et al., 2008; Bladen et al., 2014). For instance, the scorpion peptide Kurtoxin accelerates deactivation of T-type and L-type Ca_v_ channels, slows deactivation of P-type calcium channels, with little to no alteration of N-type Ca_v_ channel kinetics. In contrast, at Na_v_ channels it slows inactivation of Na_v_1.2 and Na_v_1.5 currents thus differentially modifying the gating of individual channel isoforms (Chuang et al., 1998; Olamendi-Portugal et al., 2002; Sidach and Mintz, 2002). Thus peptide interactions with conserved binding pockets on Na_v_/Ca_v_ channel voltage sensor domains can cause distinct changes to various biophysical channel parameters. Examination of other VSD Na_v_/Ca_v_ cross-interacting peptides may help identify the molecular determinants of such polypharmacology.

### Pn3a Potency Against Rat DRG Neuron I_Na_ and Recombinant hNa_v_1.7

The apparent affinity of Pn3a for small-diameter DRG neuronal *I*_*Na*_ is ∼250 nM (Figure 1D), or ∼250-fold less potent than reported for recombinant hNa_v_1.7 channels (Deuis et al., 2017). This is likely due to contribution from Pn3a-insensitive Na_v_ isoforms that also mediate DRG-*I*_*Na*_, but may also depend on orthologue differences (mouse, rat vs human), and/or dissimilar experimental conditions (recording solutions, pulse protocols, analysis criteria, etc). We evaluated the potency of Pn3a against recombinant hNa_v_1.7 using the same solutions and pulse protocols applied in our recordings of rat DRG neurons (Supplementary Figure S3). Under our experimental condition, Pn3a inhibition of hNa_v_1.7 channels stably expressed in CHO cells was reliably distinguished from current rundown at concentrations >10 nM (Supplementary Figure S1A) and became virtually irreversible at higher concentrations (Supplementary Figure S1B). We estimated *IC*_*50*_s of 31.6 ± 1.0 nM (V_h_ = −80 mV, *n* = 5) and 15.9 ± 0.4 nM (V_h_ = −120 mV, *n* = 5) for Pn3a inhibition of hNa_v_1.7 mediated currents (Supplementary Figure S1C). It has been reported that Pn3a block reaches steady-state after “tens to hundreds of seconds” of exposure (Deuis et al., 2017), thus we surmise that the ∼30-fold difference in potency reported by us may arise from differences in experimental approaches.

From our analysis, a ∼10-fold higher does Pn3a potency against recombinant Na_v_1.7 than small-diameter DRG neuron *I*_*Na*_ was observed, which is indicative of a large contribution from Pn3a-insensitive Na_v_ isoforms to the total DRG neuron Na^+^ current. This implies that the sole inhibition of Na_v_1.7 channels would have limited “hyperexcitabilty attenuation” potential in these cells.

### Potential Mechanism of Pn3a/Opioid Antinociception

Previous studies showed that Pn3a was antinociceptive when sub-therapeutic concentrations of Pn3a was applied in combination with sub-therapeutic opioid doses. A mechanism for such Pn3a/opioid antinociceptive “synergism” could not be defined as OR modulators had no effects on Na_v_1.7 nor does Pn3a treatment appear to alter OR signaling (Deuis et al., 2017; Mueller et al., 2020).

Consistently, Pn3a treatment was ineffective in rodent models of acute nociception or inflammatory pain, also in agreement with studies where other Na_v_1.7 modulators were assayed such as the VSD-peptide ProTx-II (Schmalhofer et al., 2008) and small molecule PF-04856264 (Deuis et al., 2017). The overall contribution of Na_v_1.7 to pain in some animal behaviour-based models has been questioned (Shields et al., 2018), whereas research and development of multiple potent and selective Na_v_1.7 inhibitors have failed to recapitulate the pain-free state that characterized CIP in humans (Eagles et al., 2020). Importantly, the genetic ablation of *SCN9*A in mice and in a human CIP patient, the absence of functional Na_v_1.7 leads to increases in endogenous opioid-dependent analgesia and diminished pain-induced peripheral nociceptive drive (Minett et al., 2015). In the same IB_4_^−^ DRG afferent population, Pn3a suppresses HVA *I*_*Ca*_ with an IC_50_ of ∼6 μM, which is ∼20-fold less potent than toward *I*_*Na*_. Post-surgical additive antinociception was verified by local co-administration of Pn3a with sub-therapeutic oxycodone or baclofen (Mueller et al., 2019). These two compounds are agonists of the opioid and GABA_B_ receptors (respectively), whose activation leads to G protein signaling cascades that ultimately decrease neuronal HVA Ca_v_ currents (Hescheler et al., 1987; Page et al., 2006; Sadeghi et al., 2017). The antinociceptive effects of OR agonists such as DAMGO and morphine are enhanced in the presence of Ca_v_1 and Ca_v_2.2 channel inhibitors (Contreras et al., 1988; Barro et al., 1995). We have shown that sub-therapeutic Pn3a when co-applied with the OR activator (DAMGO, Figure 4A) additively inhibit HVA *I*_*Ca*_, but not with the OR antagonist (naloxone, Figure 4B). With the exception of Ca_v_2.3, Pn3a equipotently inhibited all HVA-Ca_v_s in heterologous expression experiments (Figure 2). Altogether, HVA Ca_v_’s important roles in neuroexcitability, abundant expression, their inhibition by downstream activation of GPCRs and the hereby demonstrated sensitivity to Pn3a are consistent with decreased perception of painful stimuli by sensory neurons at the local and systemic doses reported previously. Hence providing a plausible mechanism for antinociceptive effects observed in various animal pain models as opposed to the exclusive inhibition of Na_v_1.7 channels.

## Conclusion

Pn3a inhibits HVA *I*_*Ca*_ in small diameter IB_4_^−^ DRG neurons and suppresses recombinant L-, P/Q-, N-, but not R-type Ca_v_ channels. Pn3a inhibits Ca_v_2.2 mediated currents by promoting a hyperpolarizing shift in SSI without affecting activation. Finally, Pn3a inhibition of neuronal *I*_*Ca*_ is enhanced by opioid receptor activation. Compounded inhibition of afferent HVA Ca^2+^ currents represents a plausible mechanism behind the antinociceptive effects observed in rodents treated with Pn3a and sub-therapeutic *µ*-opioid receptor agonists.

## Data Availability Statement

The raw data supporting the conclusions of this article will be made available by the authors, without undue reservation.

## Ethics Statement

The animal study was reviewed and approved by University of Sydney.

## Author Contributions

JM, NM, RF-U, DA, and MC conceived and design the research. JM, NM, and RF-U performed experiments. JM, NM, and RF-U analyzed and interpreted the data. JM, DA, and MC provided reagents. All authors reviewed, revised and approved the final paper.

## Funding

This work was supported by the National Health and Medical Research Council (NHMRC) Program Grant (APP1072113) to DA and MC, and the Rebecca Cooper Foundation for Medical Research Project Grant (PG2019396) to JM, during the conduct of the study.

## Conflict of Interest

The authors declare that the research was conducted in the absence of any commercial or financial relationships that could be construed as a potential conflict of interest.
