# Supplementary figures and images for: Spider Venom Peptide Pn3a Inhibition of Primary Afferent High Voltage-Activated Calcium Channels
Source: Front Pharmacol. 2021 Jan 28;11:633679. doi: 10.3389/fphar.2020.633679 (PMC7875911; doi:10.3389/fphar.2020.633679)

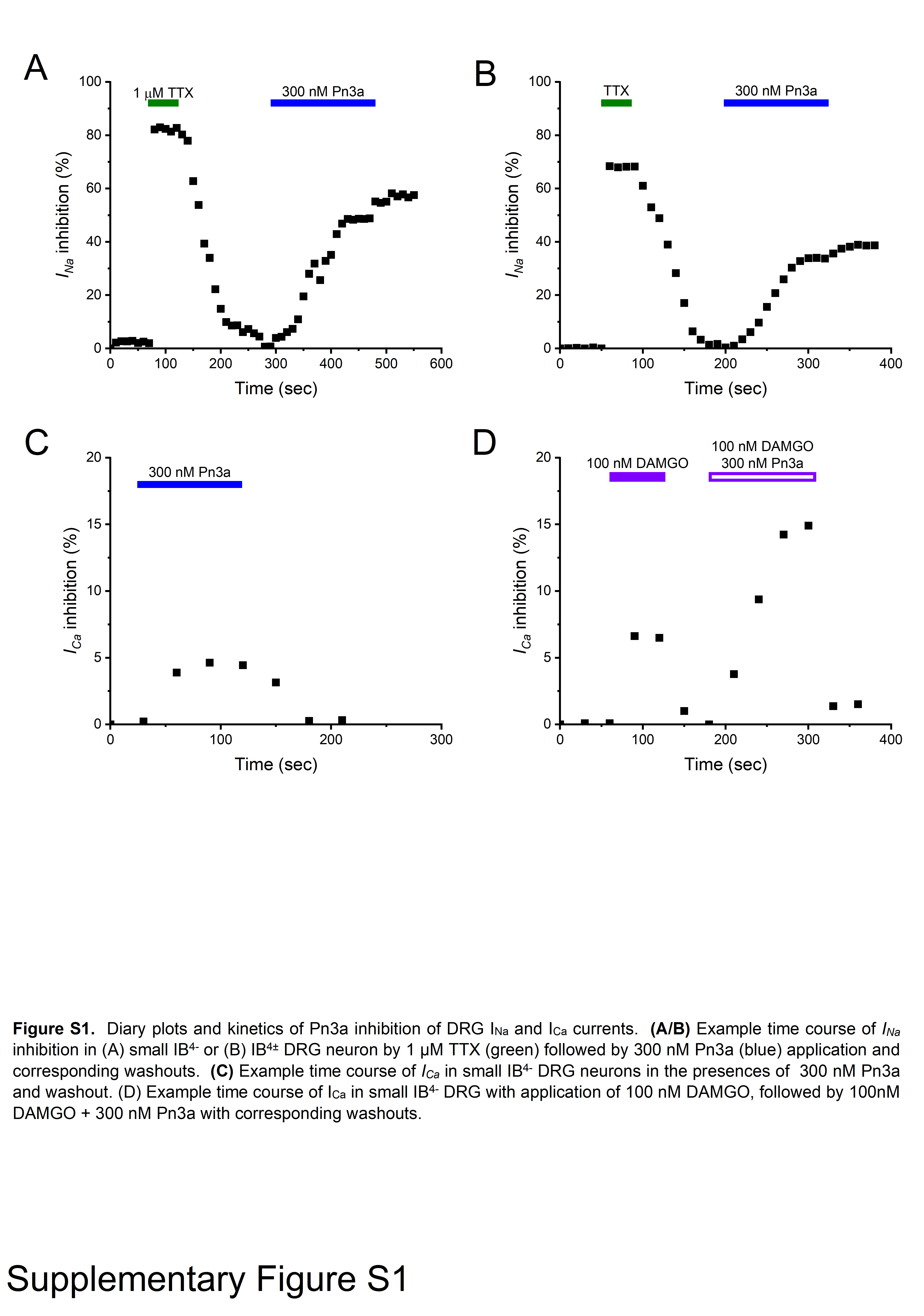

Supplement: Supplementary file 1 [file image1.tif]

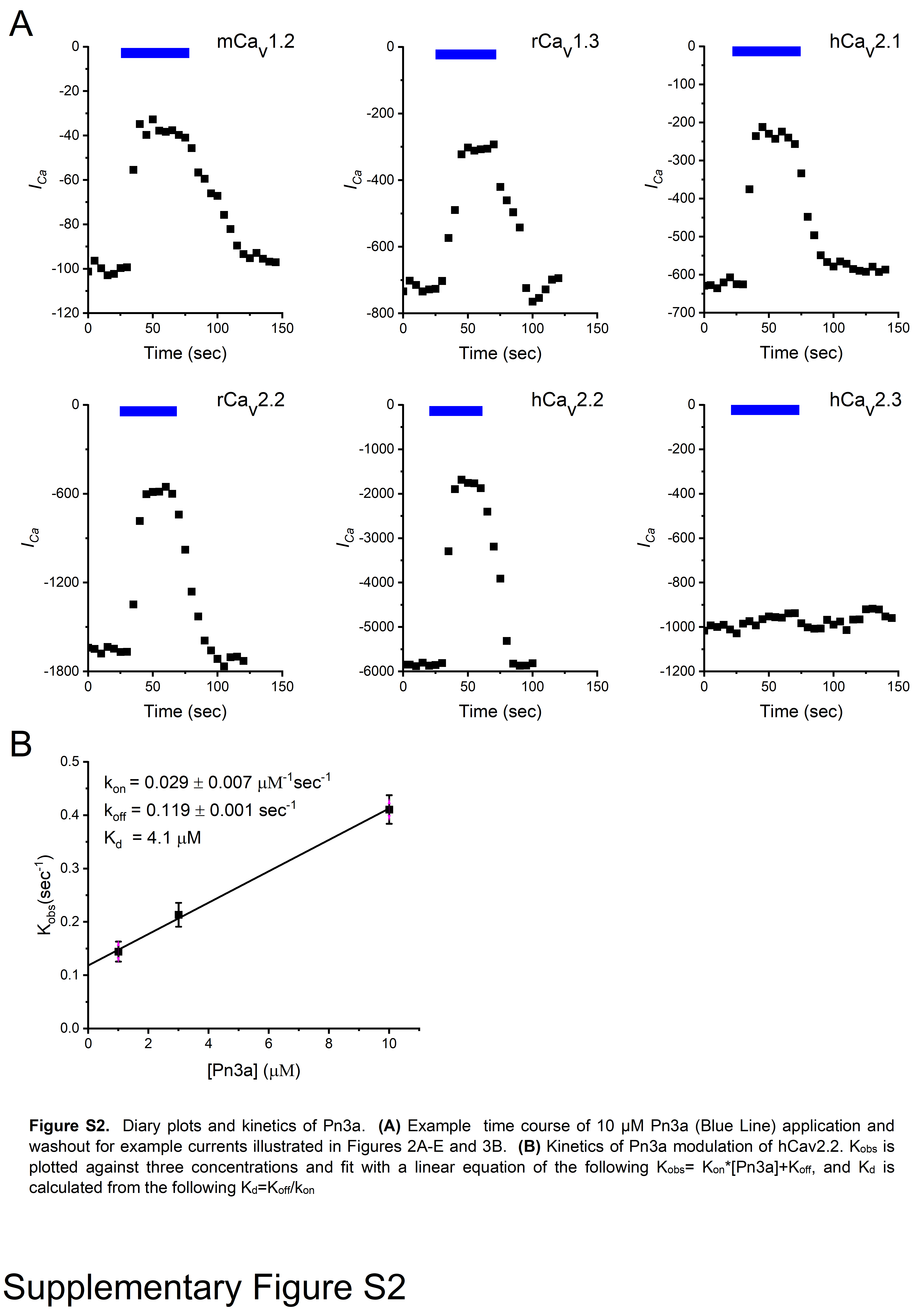

Supplement: Supplementary file 2 [file image2.tif]

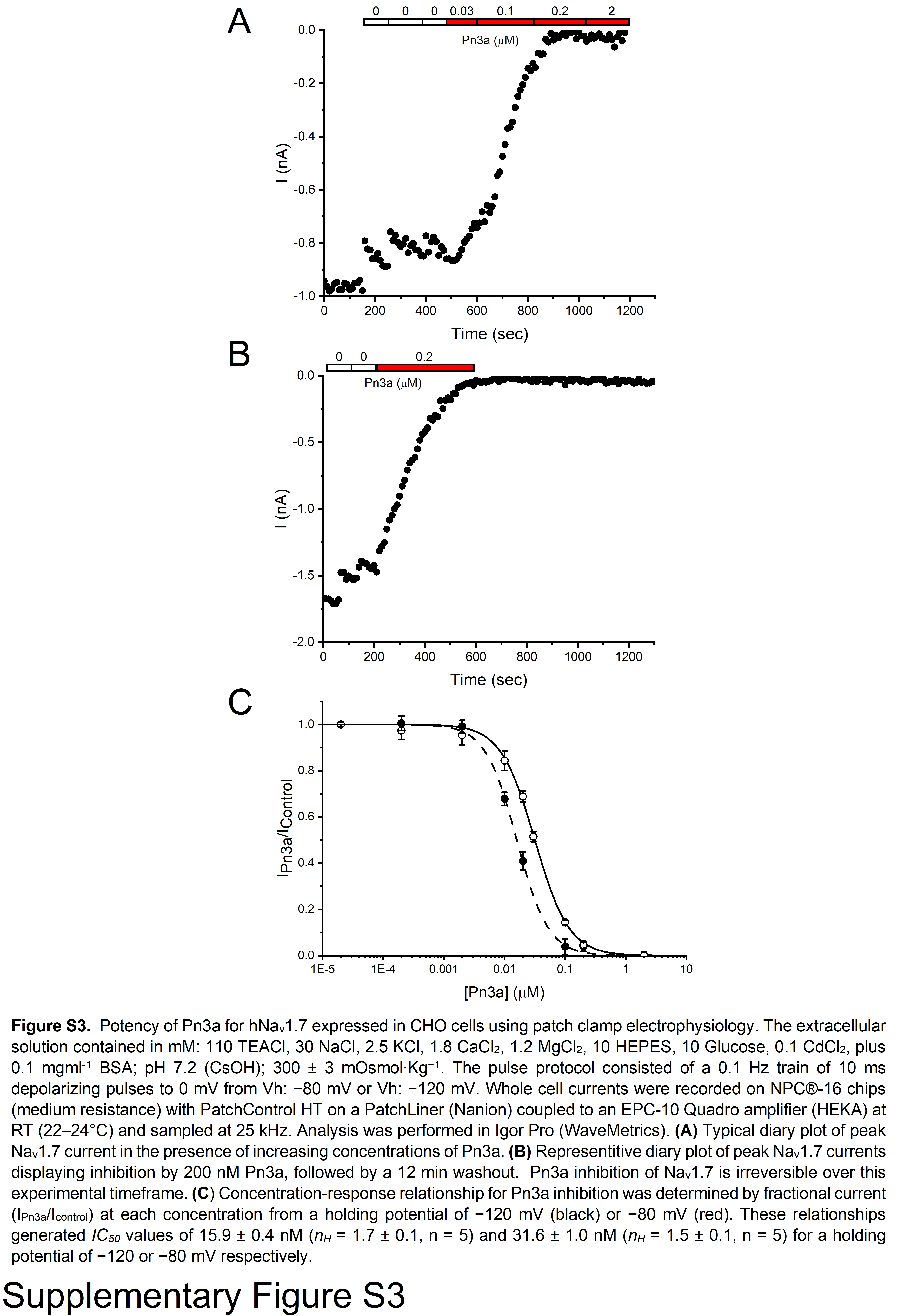

Supplement: Supplementary file 3 [file image3.tif]
